# Euglycemic Diabetic Ketoacidosis Due to Sodium–Glucose Cotransporter 2 Inhibitor Use in Two Patients Undergoing Pancreatectomy

**DOI:** 10.1089/pancan.2018.0016

**Published:** 2018-11-15

**Authors:** Devon J. Pace, Katerina Dukleska, Samantha Phillips, Vanessa Gleason, Charles J. Yeo

**Affiliations:** ^1^ Department of Surgery, Sidney Kimmel Medical College, Thomas Jefferson University, Philadelphia, Pennsylvania.; ^2^ Department of Pharmacy, Thomas Jefferson University Hospital, Philadelphia, Pennsylvania.

**Keywords:** canagliflozin, dapagliflozin, distal pancreatectomy, euglycemic DKA, pancreatic cancer, pancreaticoduodenectomy, SGLT-2 inhibitor

## Abstract

**Background:** Euglycemic diabetic ketoacidosis (euDKA) is a potential side effect associated with inhibitors of the sodium–glucose cotransporter 2 (SGLT-2). This effect is most often recognized during physiologic stress (i.e., sepsis) or in patients who undergo surgery.

**Case presentations:** Case 1: A 66-year-old woman underwent distal pancreatectomy with *en bloc* splenectomy after presenting with a biopsy-proven pancreatic adenocarcinoma in the body of the pancreas noted incidentally on a screening magnetic resonance imaging for an ovarian mass. The patient had a history of type 2 diabetes mellitus (T2DM) and used canagliflozin, which she was instructed to hold 24 h before surgery. Case 2: A 75-year-old man underwent a pylorus-preserving pancreaticoduodenectomy after presenting with obstructive jaundice. This patient also had a history of T2DM and was on dapagliflozin, which he was also instructed to hold 24 h before surgery. Postoperatively, both patients were diagnosed with euDKA, which was suspected primarily because of intraoperative and postoperative polyuria.

**Conclusions:** SGLT-2 inhibitors are associated with euDKA that can be potentiated in patients who undergo surgery. This medication side effect can be easily unrecognized and potentially lead to significant morbidity.

## Introduction

Euglycemic diabetic ketoacidosis (euDKA) is an underrecognized phenomenon associated with the use of SGLT-2 inhibitors in the perioperative period.^1^ Sodium–glucose cotransporter 2 (SGLT-2) inhibitors are oral hypoglycemic agents that promote glucosuria by inhibiting the renal glucose reabsorption in the proximal convoluted tubule.^2^ They are approved and indicated for use in patients with type 2 diabetes mellitus (T2DM) as adjunctive therapies to improve glycemic control.^3^ In this study, we present two patients who underwent a pancreatectomy for pancreatic adenocarcinoma complicated by the development of euDKA because of preoperative use of SGLT-2 inhibitors.

## Case Presentations

### Case 1

**FIG. 1.**
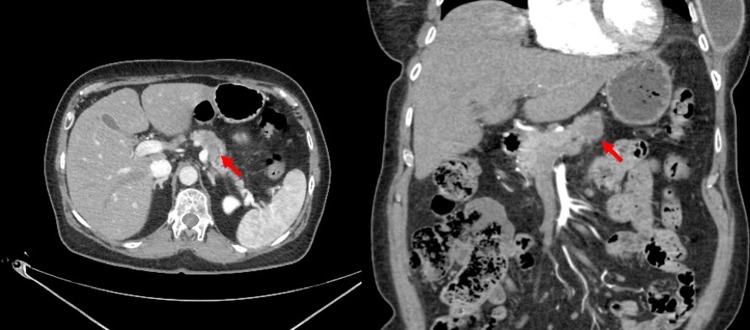
Relevant imaging for case 1: select CT imaging demonstrating a hypoattenuating and hypoenhancing lesion in the body of the pancreas (*red arrow*). CT, computed tomography.

The patient is a 66-year-old woman who initially presented with an incidental finding of a body of pancreas mass on magnetic resonance imaging for follow-up of a stable ovarian cyst. Computed tomography redemonstrated a hypodense mass in the body of the pancreas (Fig. 1). Fine-needle aspiration biopsies returned positive for pancreatic adenocarcinoma. There was no evidence of dissemination, and baseline tumor markers were within normal range. Her medical history was notable for T2DM on canagliflozin and sitagliptin. Her preoperative hemoglobin A_1c_ (HbA_1c_) was 8.2%. The patient was offered surgical resection and was instructed to hold all oral hypoglycemic agents 24 h before surgery.

She underwent an uncomplicated distal pancreatectomy with *en bloc* splenectomy. Over the first 12 h after surgery, it was noted that the patient had polyuria (urine output range: 100–325 mL/h). On routinely obtained serial laboratory analyses, the serum bicarbonate level was noted to be consistently low in the setting of anion gap and absence of lactic acidosis (Table 1).

**Table 1.** **Overview of Laboratory Trends in 6 h Increments Postoperatively for Case 1: Euglycemic Diabetic Ketoacidosis was Suspected 16 h Postoperatively**

	0 h	6 h	12 h	18 h	24 h	30 h
Serum carbon dioxide (reference range: 24–32 mmol/L)	20	16	14	15	20	20
Anion gap (reference range: 4–16 mmol/L)	16	19	20	19	10	9
β-Hydroxybutyrate (reference range: 0.2–2.8 mg/dL)	n/a	n/a	n/a	48.1	7.7	3.4
Glucose (reference range: 70–100 mg/dL)	159	165	155	224	143	153
Urinalysis (reference ranges: ketone—negative)	n/a	n/a	n/a	2+ Ketone bodies	1+ Ketone bodies	Trace ketone bodies

n/a, not available.

There was suspicion that the patient may be developing euDKA because of her use of canagliflozin. A serum β-hydroxybutyrate was obtained and it was markedly elevated at 48.1 mg/dL (reference range: 0.2–2.8 mg/dL). A urinalysis was performed that demonstrated glucosuria and ketonuria. Up to this point, the patient's serum glucose level was only modestly elevated (range: 155–224 mg/dL).

After the recognition of euDKA, an intravenous insulin infusion was initiated and the patient was fluid resuscitated. Within 10 h after such treatment, there was improvement in the β-hydroxybutyrate levels, the anion gap normalized, and the urinalysis only demonstrated trace ketone bodies (Table 1). The remainder of the patient's postoperative course was uncomplicated and she was discharged on postoperative day 5 (POD 5).

Her final pathology revealed poorly differentiated invasive ductal carcinoma, with 2 of 13 specimen lymph nodes containing metastatic cancer. The patient was educated regarding the benefit of postoperative adjuvant chemotherapy.

### Case 2

**FIG. 2.**
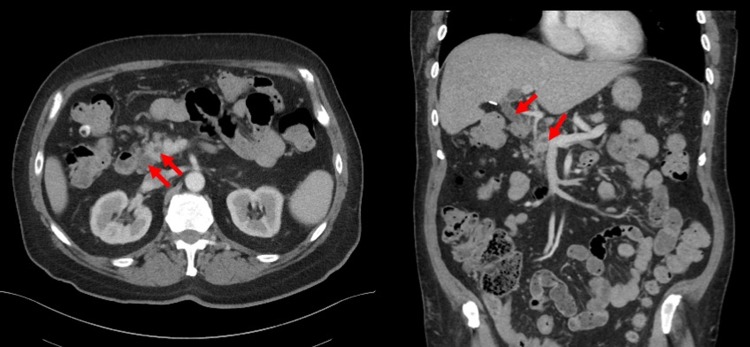
Relevant imaging for case 2: select contrast-enhanced CT imaging demonstrating a double duct sign (*red arrow*) where both the common bile duct and main pancreatic duct are simultaneously visualized and dilated.

The patient is a 75-year-old man who initially presented with obstructive jaundice and elevated liver function tests. He underwent an endoscopic ultrasound, which demonstrated a mass in the head of the pancreas with an associated bile duct stricture. Endoscopic retrograde cholangiography was performed, with biliary endoprosthesis placement. He had no evidence of metastatic disease on axial imaging, and although there was no identifiable pancreatic mass, the patient did have a double duct sign (Fig. 2). His medical history was notable for T2DM on dapagliflozin, glipizide, metformin, and liraglutide. His preoperative HbA_1c_ was 7.3%.

The patient was offered surgical resection and was instructed to hold all oral hypoglycemic agents 24 h before surgery. He underwent an uncomplicated pylorus preserving pancreaticoduodenectomy. Over the first 12 h after surgery, it was noted that the patient had polyuria (urine output range: 150–300 mL/h). On routinely obtained serial laboratory analysis, the serum bicarbonate level was noted to be consistently low in the setting of a normal anion gap and absence of a lactic acidosis (Table 2).

**Table 2.** **Overview of Laboratory Trends in 6 h Increments Postoperatively for Case 2: Euglycemic Diabetic Ketoacidosis was Suspected 16 h Postoperatively**

	0 h	6 h	12 h	18 h	24 h	30 h
Serum carbon dioxide (reference range: 24–32 mmol/L)	17	16	15	16	15	22
Anion gap (reference range: 4–16 mmol/L)	15	15	14	19	14	9
β-Hydroxybutyrate (reference range: 0.2–2.8 mg/dL)	31.8	36.2	42.8	50.8	14.3	8.9
Glucose (reference range: 70–100 mg/dL)	158	173	169	225	182	164
Urinalysis (reference range: ketone—negative)	n/a	n/a	n/a	3+ Ketone bodies	1+ Ketone bodies	Negative for ketone bodies

Retrospectively obtained β-hydroxybutyrate levels were elevated immediately postoperatively, despite a normal anion gap.

There was suspicion that the patient may be developing euDKA because of his use of dapagliflozin. A serum β-hydroxybutyrate was obtained and it was markedly elevated at 50.8 mg/dL (reference range: 0.2–2.8 mg/dL). It was only at that time that the patient had an abnormal anion gap of 19 mmol/L. A urinalysis was performed that demonstrated glucosuria and ketonuria. Up to this point, the patient's serum glucose level was only modestly elevated (range: 158–225 mg/dL). β-Hydroxybutyrate levels were retrospectively obtained on POD 1 by analyzing samples that were routinely collected on POD 0, which demonstrated that they were consistently elevated postoperatively in the setting of a normal anion gap.

After the recognition of euDKA, an intravenous insulin infusion was initiated and the patient was fluid resuscitated. Within 12 h after such treatment, there was improvement in the β-hydroxybutyrate levels, the anion gap normalized, and the urinalysis was negative for ketone bodies. The remainder of the patient's postoperative course was uncomplicated and he was discharged on POD 6.

His final pathology revealed moderately differentiated pancreatic ductal adenocarcinoma, with metastatic carcinoma identified in 2 of 14 specimen lymph nodes. He was also educated regarding the benefits of postoperative adjuvant chemotherapy.

## Conclusions

Pancreatectomy is a complex abdominal procedure associated with numerous complications in up to 50% of patients, and mortality in 0.5–5% of patients. Common complications of pancreatectomy include delayed gastric emptying, pancreatic fistula formation, hemorrhage, intraabdominal abscess, and many others.^4^ Uncommon findings of postpancreatectomy are polyuria, glucosuria, ketonuria, and acidosis. Therefore, when present, these findings should alert clinicians to investigate the causes unrelated to surgery. The two patients discussed previously manifested these uncommon postpancreatectomy findings and, to our knowledge, these are the first reports to describe euDKA in patients who underwent pancreatectomy as a consequence of SGLT-2 inhibitor use.

euDKA was first described in 1973 in a report that described a series of 37 episodes of severe ketosis in patients with normal or modestly elevated serum glucose levels.^5^ This condition continues to be a diagnostic challenge because the euglycemia oftentimes obscures the underlying ketosis. SGLT-2 inhibitors are a class of oral hypoglycemics that have been shown to improve glycemic control in patients with T2DM.^3^ However, in 2015, the Food and Drug Administration released a communication describing the risk of euDKA associated with SGLT-2 inhibitors.^1^ This risk is increased in the setting of physiologic stress and surgery that has resulted in the recommendation for these medications to be held 24–72 h preoperatively.^2,6^ Ketosis and particularly euDKA seems to specifically be associated with dual SGLT-1/2 inhibitors and SLGT-2 inhibitors. To our knowledge, none of the other patients' home medications or other oral hypoglycemics have been reported to be associated with euDKA. Of importance, other oral hypoglycemics such as glucagon-like peptide-1 receptor agonists or dipeptidyl peptidase-4 inhibitors have been associated with pancreatitis and heart failure, respectively.^7,8^ Therefore, it is important for clinicians to be aware of these rare but important serious medication side effects.

**FIG. 3.**
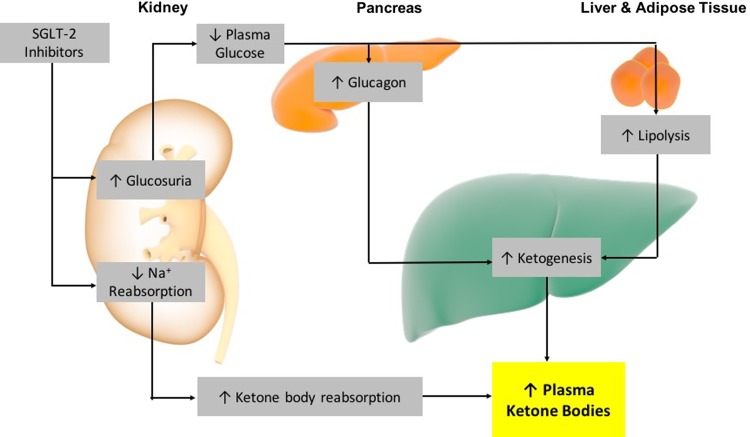
Proposed mechanism for the development of euglycemic diabetic ketoacidosis in patients exposed to SGLT-2 inhibitors. SGLT-2, sodium–glucose cotransporter 2.

The mechanism of action for SGLT-2-induced euDKA is attributed to increased lipolysis and fatty acid oxidation because of a reduced plasma insulin-to-glucagon ratio, resulting in increased ketone body production and increased ketone body reabsorption in the kidney (Fig. 3).^2,9^ This, along with the metabolic stress of surgery combined with prolonged fasting, increases the risk of SGLT-2-induced euDKA.^2,10^ In this case series, we present two patients with euDKA associated with canagliflozin and dapagliflozin use, and a delay in recognition. In part, this delay was because of normal glucose levels and in the second case, an initially normal anion gap.

To avoid this complication, we recommended that SGLT-2 inhibitors be held at least 5 days preoperatively. Based upon the relatively long half-life of these medications (11–13 h), the drug effect should be absent after 5 days and the risk of euDKA should be reduced. Clinicians must also be mindful that the half-life of SGLT-2 inhibitors can be prolonged in patients with diminished renal function, and the drug effect in this instance may still be present even after the medication is held for 5 days.^2^ These cases underscore the importance of a having a high clinical suspicion for euDKA in patients who are taking SGLT-2 inhibitors who undergo surgery, even in the absence of an anion gap.

## Abbreviations Used

CT: computed tomography
euDKA: euglycemic diabetic ketoacidosis
HbA_1c_: hemoglobin A_1c_
SGLT-2: sodium-glucose cotransporter 2
T2DM: type 2 diabetes mellitus

## References

[1] Food and Drug Administration (FDA). FDA Drug Safety Communication: FDA warns that SGLT2 inhibitors for diabetes may result in a serious condition of too much acid in the blood. Issued 5 15, 2015.

[2] Milder DA, Milder TY, Kam PCA Sodium-glucose co-transporter type-2 inhibitors: pharmacology and peri-operative considerations. Anaesthesia. 2018;73:1008–1018 29529345 10.1111/anae.14251

[3] Molugulu N, Yee LS, Ye YT, et al. Systematic review of metformin monotherapy and dual therapy with sodium glucose co-transporter 2 inhibitor (SGLT-2) in treatment of type 2 diabetes mellitus. Diabetes Res Clin Pract. 2017;132:157–168 28797524 10.1016/j.diabres.2017.07.025

[4] Winter JM, Cameron JL, Campbell KA, et al. 1423 pancreaticoduodenectomies for pancreatic cancer: a single-institution experience. J Gastrointest Surg. 2006;10:1199–1210; discussion 1210–1211. 17114007 10.1016/j.gassur.2006.08.018

[5] Munro JF, Campbell IW, McCuish AC, et al. Euglycaemic diabetic ketoacidosis. Br Med J. 1973;2:578–580 4197425 10.1136/bmj.2.5866.578 PMC1592207

[6] Handelsman Y, Henry RR, Bloomgarden ZT, et al. American Association of Clinical Endocrinologists and American College of Endocrinology position statement on the association of SGLT-2 inhibitors and diabetic ketoacidosis. Endocr Pract. 2016;22:753–762 27082665 10.4158/EP161292.PS

[7] Egan AG, Blind E, Dunder K, et al. Pancreatic safety of incretin-based drugs—FDA and EMA assessment. N Engl J Med. 2014;370:794–797 24571751 10.1056/NEJMp1314078

[8] Food and Drug Administration (FDA). FDA drug safety communication: FDA adds warnings about heart failure risk to labels of type 2 diabetes medicines containing saxagliptin and alogliptin. 2016

[9] Qiu H, Novikov A, Vallon V Ketosis and diabetic ketoacidosis in response to SGLT2 inhibitors: basic mechanisms and therapeutic perspectives. Diabetes Metab Res Rev. 2017;33:e2886 10.1002/dmrr.2886 28099783

[10] Blau JE, Tella SH, Taylor SI, et al. Ketoacidosis associated with SGLT2 inhibitor treatment: analysis of FAERS data. Diabetes Metab Res Rev. 2017;33. doi:10.1002/dmrr.2924 PMC5950709 28736981

